# Using Bayesian time-stratified case-crossover models to examine associations between air pollution and “asthma seasons” in a low air pollution environment

**DOI:** 10.1371/journal.pone.0260264

**Published:** 2021-12-08

**Authors:** Matthew Bozigar, Andrew B. Lawson, John L. Pearce, Erik R. Svendsen, John E. Vena

**Affiliations:** Department of Public Health Sciences, Medical University of South Carolina, Charleston, South Carolina, United States of America; Valahia University of Targoviste, ROMANIA

## Abstract

Many areas of the United States have air pollution levels typically below Environmental Protection Agency (EPA) regulatory limits. Most health effects studies of air pollution use meteorological (e.g., warm/cool) or astronomical (e.g., solstice/equinox) definitions of seasons despite evidence suggesting temporally-misaligned intra-annual periods of relative asthma burden (i.e., “asthma seasons”). We introduce asthma seasons to elucidate whether air pollutants are associated with seasonal differences in asthma emergency department (ED) visits in a low air pollution environment. Within a Bayesian time-stratified case-crossover framework, we quantify seasonal associations between highly resolved estimates of six criteria air pollutants, two weather variables, and asthma ED visits among 66,092 children ages 5–19 living in South Carolina (SC) census tracts from 2005 to 2014. Results show that coarse particulates (particulate matter <10 μm and >2.5 μm: PM_10-2.5_) and nitrogen oxides (NO_x_) may contribute to asthma ED visits across years, but are particularly implicated in the highest-burden fall asthma season. Fine particulate matter (<2.5 μm: PM_2.5_) is only associated in the lowest-burden summer asthma season. Relatively cool and dry conditions in the summer asthma season and increased temperatures in the spring and fall asthma seasons are associated with increased ED visit odds. Few significant associations in the medium-burden winter and medium-high-burden spring asthma seasons suggest other ED visit drivers (e.g., viral infections) for each, respectively. Across rural and urban areas characterized by generally low air pollution levels, there are acute health effects associated with particulate matter, but only in the summer and fall asthma seasons and differing by PM size.

## 1.0 Introduction

Emergency department (ED) visits for asthma have complex drivers influenced by disease severity, access to and utilization of various preventive care services [1], and numerous environmental factors [2]. Asthma exacerbations and ED visits have been associated with multiple ambient air pollutants, complex mixtures, and temporal lags [3–6]. While there is evidence of seasonality, previous studies have used several common season definitions, such as astronomical seasons defined by equinoxes and solstices (i.e., winter, spring, summer, fall), meteorological seasons (e.g., cool, warm), and 3-month simplifications (e.g., winter: December January, and February) to name a few. When exacerbations were linked with non-environmental factors, such as body mass index (BMI), researchers employed traditional astronomical seasons [7]. In studies focused on ambient air pollution exposures, scholars tend to define time periods by warm and cold seasons, reflective of temporal patterns in the exposures [8–10]. In addition, some acute health effects of air pollution studies used simplified 3-month seasonal blocks [11, 12]. Air pollution, pollen, and viruses are several examples of seasonally-varying environmental exposures exacerbating asthma symptoms [13–17]. Some of these exposures may co-occur over time, while others may only partially overlap, if at all. Viral infections tend to increase in cooler periods such as the fall and winter [18]. Seasonal associations between rain events or thunderstorms and asthma exacerbations have been found in warmer months, such as the spring and summer in the US, with a likely mechanism being the release of grass pollen [19–21].

Air pollution and other place-based ambient environmental factors can interact in complex ways among people with asthma [2]. Most short-term analyses of air pollution and asthma ED visits focus on highly urbanized areas [8, 10, 22, 23] that tend to have higher air pollution levels than suburban or rural areas and are monitored more thoroughly [24]. Acute health effects studies of air pollution have been predominantly designed to estimate associations and dose-response relationships in such urban environments. They assume that temporal variation in asthma ED visits are driven by seasonal patterns among ambient pollutants, themselves, despite evidence of temporally-misaligned patterns in the asthma ED visits. At the national level, aggregated asthma ED visits peak in the fall, but there are differing regional and local seasonal burdens [25–27]. Thus, study type, objectives, location, exposures, exposure levels, and exposure timing are elements that should be considered when defining seasons for an asthma outcome, such as emergency department visits, that can exhibit large temporal differences within a given year.

Though controlled exposure studies of air pollutants are important for understanding disease pathways [28, 29], they are ethically untenable for a relatively severe outcome such as ED visits. As such, epidemiologists must contend with various observational designs that attempt to infer disease patterns in study populations. Case-crossover studies inherently control for time-invariant characteristics and are therefore useful for studying short-term, time-varying exposures affecting health [30]. They are additionally useful when only case data are known, such as in administrative health datasets (e.g., ED visits), in which there are no contrasting non-case events to provide outcome variability to develop a data model. With care, case-crossover models can be developed for individuals in case-only datasets because non-case events (i.e., referents) can be strategically selected and temporally matched to the case events for each person. Case-crossover designs do not need to be aggregated over space and therefore permit spatially explicit exposure estimates, unlike time-series designs [31]. While time series designs serve as a primary option for case-only data in environmental epidemiology, they apply only one exposure estimate to all study participants in each time period. Bayesian case-crossover models have been equally or more accurate than frequentist versions [32], and are attractive for their robustness to model misspecification, efficiency, flexibility, and the ability to include informative prior information. Despite their potential, at the time of writing we found only two studies led by Li et al. (2013) and Guo et al. (2014) that had developed Bayesian case-crossover models for studies of acute health effects of air pollution [32, 33].

According to the US Environmental Protection Agency (EPA), annual average particulate matter <2.5 μm (PM_2.5_) levels are deemed “safe” when they are below 12 μg/m^3^ [34]. For particulate matter <10 μm (PM_10_), the average annual safety standard is 50 μg/m^3^ [34]. Many rural areas with low air pollution levels (below these EPA regulatory limits) can have a relatively high burden of asthma ED visits [35–37], but the environmental drivers of asthma ED visits across urban-rural areas are understudied. In addition, few researchers have reconsidered seasonal definitions to focus on the intra-annual periods of relative burden, “asthma seasons”, and the unique ambient environmental drivers of those burdens across urban-rural subpopulations.

Our specific objectives are to 1) detail trends in pediatric asthma ED visits in a large and diverse study population and geography, and subsequently to 2) define asthma seasons to identify ambient air pollutants associated with seasonal burdens. We hypothesize that associations between ambient air pollution and asthma ED visits vary by specific asthma seasons in a low air pollution environment. To address our hypothesis, we estimate associations in South Carolina’s (SC) asthma seasons between EPA criteria air pollutants, weather, and asthma ED visits for children living in South Carolina from 2005 to 2014 using a Bayesian time-stratified case-crossover design.

## 2.0 Methods

### 2.1 Health outcome

This research was approved (Pro00068172) by the Medical University of South Carolina institutional review board as a part of the SocioEnvironmental Associations with Asthma Increased Risk (SEA-AIR) study. Consent for study participation was not required as the health outcomes used in this study were obtained as secondary, anonymized administrative health data. The health outcome data consisted of 66,092 ED visits with a primary diagnosis of asthma (International Classification of Disease 9, ICD9, codes 493.XX) among children ages 5–19 years residing in South Carolina from 2005 to 2014. The South Carolina Revenue and Fiscal Affairs (SCRFA) office linked records from multiple payor sources. To the best of our knowledge, the data have population wide coverage, capturing all pediatric ED visits for asthma in SC during the 10-year study period. Basic demographic information, diagnostic codes, dates of admittance and discharge, and geographic identifiers were included. Records included geographic identifiers of both ZIP codes and census tracts. Records with missing census tract identifiers (>20%) were assigned to a census tract using a novel geographic identifier assignment algorithm [38]. ED records were assigned exposure and weather estimates of their billing code census tract, respectively.

There are 1,103 census tracts in SC (2010 US Census geography), and 1,085 of them are not water-only (i.e., off the coast) or institutional-only (e.g., correctional facility) [39]. The children in this study lived in 1,079 of these 1,085 regular census tracts. Census tracts in SC average 70.6 sq km, or approximately half of a 12 by 12 km (144 sq km) grid cell utilized by the EPA Community Multiscale Air Quality (CMAQ) model [40]. However, census tracts vary widely in size because they are proportional to population density, as evidenced by their range in SC from 0.42 to 819 sq km with a standard deviation of 106.8 among the 1,085 regular census tracts.

### 2.2 Air pollutant estimates

Estimates of air pollutant exposure were “fused” from a chemical transport model, the CMAQ model, and monitored values at a 12 km resolution for the US by other researchers [40, 41]. Daily estimates were available for six EPA criteria pollutants: carbon monoxide (CO), nitrogen oxides (NO_x_), ozone (O_3_), and sulfur dioxide (SO_2_), PM_2.5_, and PM_10_. We included NO_x_ over nitrogen dioxide (NO_2_) because it incorporated both nitrogen oxides (NO and NO_2_) that have been previously linked with asthma [42]. Furthermore, we calculated the fraction of coarse particulate matter (PM_10-2.5_) by subtracting the PM_2.5_ estimates from the PM_10_ estimates, which we included in statistical modeling instead of PM_10_ that incorporates fine particulates as well.

Using ArcGIS (Environmental Systems Research Institute, Redlands, CA), the national daily gridded air pollutant estimates were first clipped to a grid encompassing SC and a surrounding 12 km buffer extending into neighboring North Carolina (NC) and Georgia (GA) to leverage nearby grid points across state lines. The daily gridded estimates were then spatially interpolated to population weighted census tracts in SC using inverse distance weighting (IDW). While estimates interpolated from a 12 km grid were likely spatially smoothed by this procedure, IDW estimates have previously been employed for the purpose of capturing temporal (i.e., daily) variation [43], which is consistent with the objectives of our acute health effects study design.

### 2.3 Weather estimates

Air temperature and dewpoint temperature data were obtained from the PRISM Climate Group in the form of daily national smooth surfaces [44]. The daily national surfaces were first clipped to the spatial extent or SC, and we then calculated the daily spatial average for each census tract by block kriging. Temporal trends in census tract air pollution and weather estimates were assessed using box and whisker plots by season over time. Seasonal correlation patterns among air pollutants were assessed by collapsing air pollution and weather estimates over the entire study period by day of the year, sub-setting by season, calculating Spearman correlations for estimate rankings, and visualizing in the form of heat maps.

### 2.4 Case and referent window selection

We used a time-stratified case-crossover design. Time-stratification of case events by year, month, and day of the week helps control for short-term, seasonal, and long-term temporal trends [32, 45]. Relative to other strategies, time-stratification has been shown to be the least biased referent selection strategy in case-crossover models because of lower time trend and “overlap” biases, respectively [46, 47]. We calculated a 3-day moving average (3DMA) over lag days 0 (day of), 1 (1 day prior), and 2 (2 days prior) for each of the pollutant exposures and the weather variables for every ED visit (i.e., case), respectively [8, 48, 49]. These 3DMAs represented the case windows. 3DMA referent windows were created by matching to each 3DMA case window on year, month, and day of the week, per the time-stratified design. If a 3DMA case window had more than three separate 3DMA referent window matches available (depending on the count of a particular day of week within a given month), we randomly sampled three 3DMA referent windows from those available. For example, the 3DMAs for respective pollutants and weather factors at the respective patient’s billing address for a hypothetical pediatric asthma ED visit on the second Tuesday of February in 2010 (case window) would be matched to three of the 3DMAs for the remaining Tuesdays in February of 2010 (referent windows). The 3DMA referent windows provided contrasts to the 3DMA case windows for times when each child was not admitted to the ED for asthma, respectively.

### 2.5 Asthma seasons

To differentiate local asthma seasons from each other and from other commonly-used definitions (e.g., astronomical seasons), we graphed individual ED visits over time. To further elucidate intra-annual patterns indicative of relative ED visit burdens, we collapsed all ED visits over 2005–2014 ED by day of the year into a single graph. Visual patterns in the graphs were used to identify short-term and long-term trends, and seasonal means were also calculated.

### 2.6 Statistical analysis

Our Bayesian hierarchical model (BHM) framework was constructed as follows:

$$y_{ij}∼Bernoulli(\pi_{ij})$$

$$logit(\pi_{ij})=\alpha +d_{c}+\beta_{p}^{\prime }X_{ij}+\beta_{w}^{\prime }W_{ij}$$ (1)

$$\left(\beta_{*}|\tau_{*})∼Normal(0,\tau_{*}^{-1}\right)$$

$$\tau_{*}∼Gamma(2,1)$$

$$\left(\alpha |\tau_{\alpha })∼Normal(0,\tau_{\alpha }^{-1}\right)$$

$$\tau_{\alpha }=\frac{1}{{sd\alpha }^{2}}$$

sdα∼uniform(0,4)

$$\left(d_{c}|\tau_{d})∼Normal(0,\tau_{d}^{-1}\right)$$

$$\tau_{d}∼Gamma(2,0.5)$$

in which the log of the probability (*π*_*ij*_) of an ED visit (*y*_*ij*_) was modeled for *i* = 1,2,…66,092 ED visit records and *j* = 1,2,3,4 case/referent windows for each record. The conditional logistic regression model (Eq 1) at the second level of the hierarchy, included an intercept (*α*), and a set of beta coefficients (***β***_*p*_) for each criteria pollutant (***X***_*ij*_), respectively. A set of respective beta coefficients (***β***_*w*_) for air temperature, dewpoint temperature, and their interaction (***W***_*ij*_) were also included. In statistical modeling, including an interaction between temperature and dewpoint temperature generally improved model fit. We helped control for temporal clustering by including a random effect for each unique year-month-day combination (*d*_*c*_) of our study period, which was indexed by *c* = 1,2,…840 combinations. The combination random effect accounted for temporal patterns such as those induced by holidays or days of the week. Coefficients and random effects were assumed to follow normal distributions, each having *τ* precision parameters that followed gamma distributions at the next level of the hierarchy, which were weakly informative [50, 51]. We sought to make few prior assumptions because the effects of low-level exposures to air pollution are lesser known. But, one of the benefits of conducting Bayesian analysis is the ability to incorporate prior information that can be leveraged in future research. To improve convergence of the *α* intercept term, another level was added to the hierarchy, allowing the precision term *τ*_*α*_ to be determined from a uniformly distributed *sda* term. Air pollutant and weather estimates were mean-centered to improve computational efficiency.

### 2.7 Model fitting and building

We fit models in the NIMBLE package in R [52–54]. NIMBLE conducts Markov chain Monte Carlo (MCMC) sampling by recompiling models written in Bayesian Using Gibbs Sampling (BUGS) language (e.g., WinBUGS) into C++ language, which greatly increases computational efficiency and stability. We fit models on all the data and season subsets, using the timing of the case day to determine the season. We removed variables that induced variable inflation due to high collinearity during model fitting. For instance, in all models CO was removed because it was highly collinear with NO_x_, likely because their main respective sources are both fuel combustion emissions [55]. In addition, CO estimates were calibrated using only one monitor in all of SC and was prone to temporal gaps [56], which may have introduced more error in its estimates relative to those of other pollutants that were monitored more comprehensively. In the overall, summer, and fall models, dewpoint temperature was removed due to its high collinearity with temperature, respectively. In the summer model, SO_2_ was additionally removed because it was highly collinear with NO_x_. We reported exponentiated *β* coefficients [57] in interquartile ranges (IQR) overall and by season, and interpreted them as the odds of an IQR increase/decrease either overall or by season (OR_IQR_), respectively. Analytic data and code to reproduce the analyses using non-identifiable data are available at https://github.com/mbozigar/asthma-seasons.

### 2.8 Sensitivity analyses

We assessed numerous ways to control for potentially unmeasured confounding factors via random effects. The inclusion of spatial (structured and unstructured) and individual (i.e., linking records over time) factors did not improve model fit by deviance information criterion (DIC). Defining seasons and strategies to control for seasonal trends in the overall model was challenging. We assessed multiple temporal cut points, indicators, non-linear effects, prior distributions, and random effects. Many referent day strategies, including unidirectional asymmetric, bidirectional symmetric, and time-stratified designs were assessed. There were minor differences across the strategies, but main findings were consistent. Results were similar for PM_10_ and PM_10-2.5_. We opted for the latter in statistical modeling to better differentiate it from PM_2.5_ and to capture changes in the PM_10-2.5_ fraction over time and space from differential local sources and contexts that have previously been found to occur in a similar geographic area [58].

## 3.0 Results

### 3.1 Descriptive results

**Fig 1:**
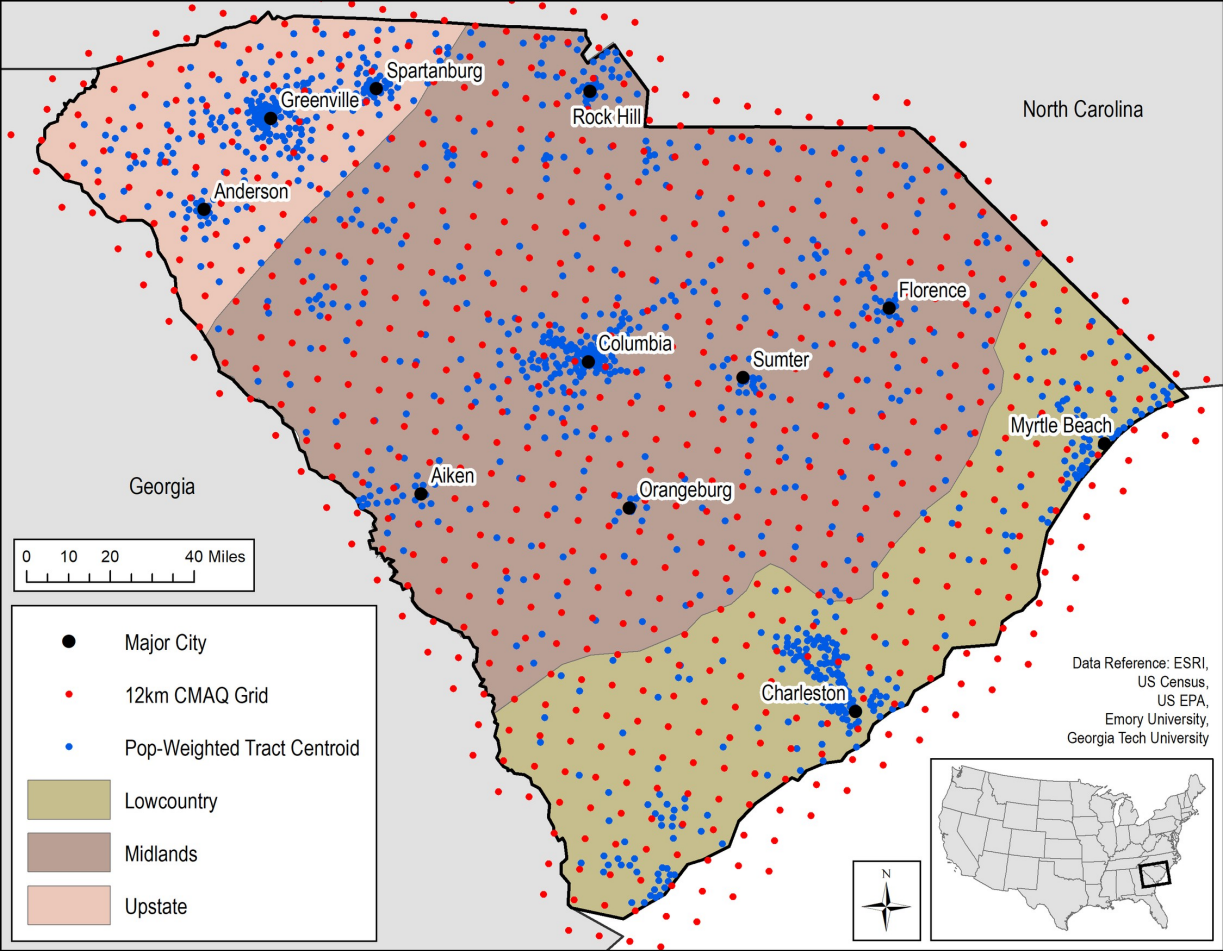
Study area of South Carolina showing its regions, main urban areas, 12 km CMAQ grid point locations, and population weighted census tract centroids (2010 US Census population estimates and geography).

Fig 1 shows the study area of SC, its regions, main urban areas, 12 km CMAQ grid point locations, and population weighted census tract centroids. Regions in SC generally delineate unique geologic and geographic features. The Lowcountry encompasses the low, coastal plain, while the Midlands region is characterized by a somewhat sandy, hilly landscape conducive to agriculture, and the Upstate consists of foothills of the Blue Ridge Mountains.

Table 1 shows the asthma ED visit and SC populations for children ages 5–19 years. Contrasted with the SC pediatric population of the same age range, the asthma ED visit population was more male (58.5 to 51.0%), younger (46.9 to 32.1%), African American (68.0 to 28.4%), and lived more in the Midlands region (52.4 to 49.9%) than other regions. The ED visit population was also predominantly on public insurance such as Medicaid (58.3%) and visited an ED in the fall (47.5%). For age-sex subgroups in the ED visit population, there were 20,034 males and 10,989 females ages 5–9 years, 11,494 males and 7,547 females ages 10–14 years, and 7,133 males and 8,895 females ages 15–19 (tabular/graphical results not shown).

**Table 1:** Population characteristics of the age 5–19 year populations that used the emergency department (ED) for asthma from 2005 to 2014 and for the state of South Carolina.

	Age 5–19 Asthma Emergency Department Visit Population	Age 5–19 South Carolina Population (2010 US Census Estimates)	
Stratum	n (frequency %)	n (frequency %)	p-Value
**Total**	66,092	921,428	N/A
**Sex**			
Male	38,661 (58.5)	470,072 (51.0)	<0.0001
Female	27,431 (41.5)	451,356 (49.0)	
**Age Group**			
5–9	31,023 (46.9)	295,850 (32.1)	<0.0001
10–14	19,041 (28.8)	297,263 (32.3)	
15–19	16,028 (24.3)	328,315 (35.6)	
**Race**			
White	17,672 (26.7)	604,871 (65.6)	<0.0001
African American	44,921 (68.0)	261,315 (28.4)	
Other	3,499 (5.3)	55,242 (6.0)	
**Payor Status**			
Public Insurance	38,516 (58.3)	Data Unavailable	N/A
Private Insurance	16,835 (25.5)	Data Unavailable	
Other	10,723 (16.2)	Data Unavailable	
**Asthma Season**			
Winter (Jan 1–Feb 28)	9,763 (14.7)	N/A	N/A
Spring (Mar 1–May 31)	17,414 (26.3)	N/A	
Summer (Jun 1–Aug 19)	7,616 (11.5)	N/A	
Fall (Aug 20 –Dec 31)	31,299 (47.5)	N/A	
**Region**			
Upstate	13,720 (20.7)	238,341 (25.9)	<0.0001
Midlands	34,616 (52.4)	459,390 (49.9)	
Lowcountry	17,756 (26.9)	223,697 (24.2)	

**Fig 2:**
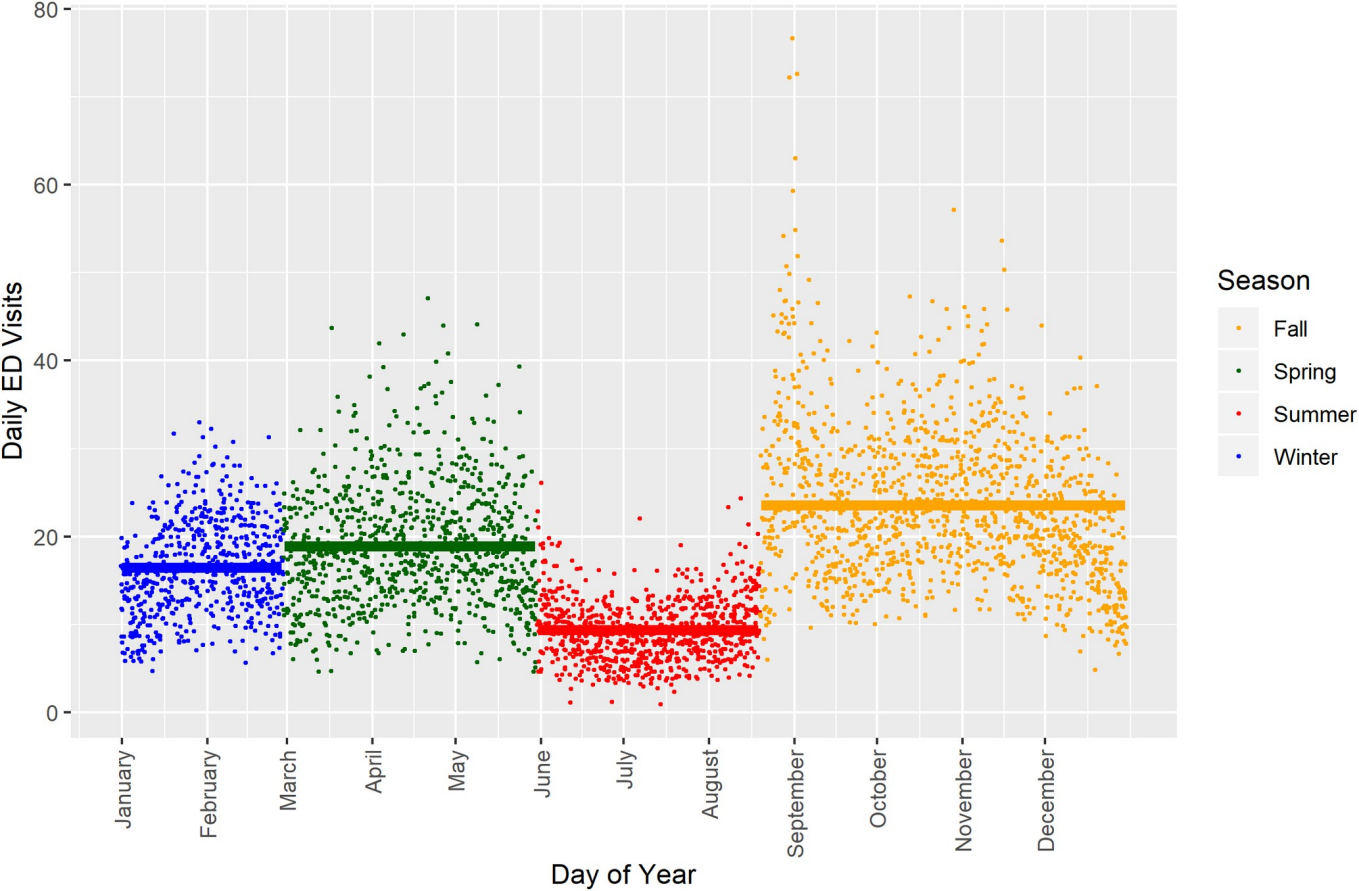
Asthma emergency department (ED) visits for ages 5–19 years from 2005 to 2014 in South Carolina grouped by admittance day of the year and asthma season (winter: January 1 –February 28/29; spring: March 1 –May 31; summer: June 1 –August 19; fall: August 20 –December 31). Seasonal mean daily ED visits were 16.5 visits/day/year in winter (medium burden), 18.9 visits/day/year in spring (medium-high burden), 9.4 visits/day/year in summer (low burden), and 23.5 visits/day/year in fall (high burden).

Fig 2 showed that ED visits may not mirror commonly used seasonal definitions, such as astronomical seasons. SC’s asthma seasons tended to start and end earlier, with the exception of winter, and were thus misaligned with astronomical seasons. We defined a medium burden (16.5 visits/day/year) winter asthma season from January 1^st^ to the end of February that paralleled the much cooler mid-winter months when children were in school. The relative increase in ED visits indicative of medium-high burden (18.9 visits/day/year) from March 1^st^ through May 31^st^, mirroring rising, albeit fluctuating, temperatures and highly variable conditions typical of the spring allergy season when children were still in school, helped us define the SC spring asthma season. The summer asthma season was characterized by low burden (9.4 visits/day/year) and an earlier June 1^st^ start and August 19^th^ end when children were generally not in school. Exhibiting the largest seasonal disparity (23.5 visits/day/year), the fall asthma season was defined as starting on August 20^th^, the approximate beginning of the school year in SC. This period begins with warm temperatures and then cools during a secondary allergy season ending December 31^st^.

**Fig 3:**
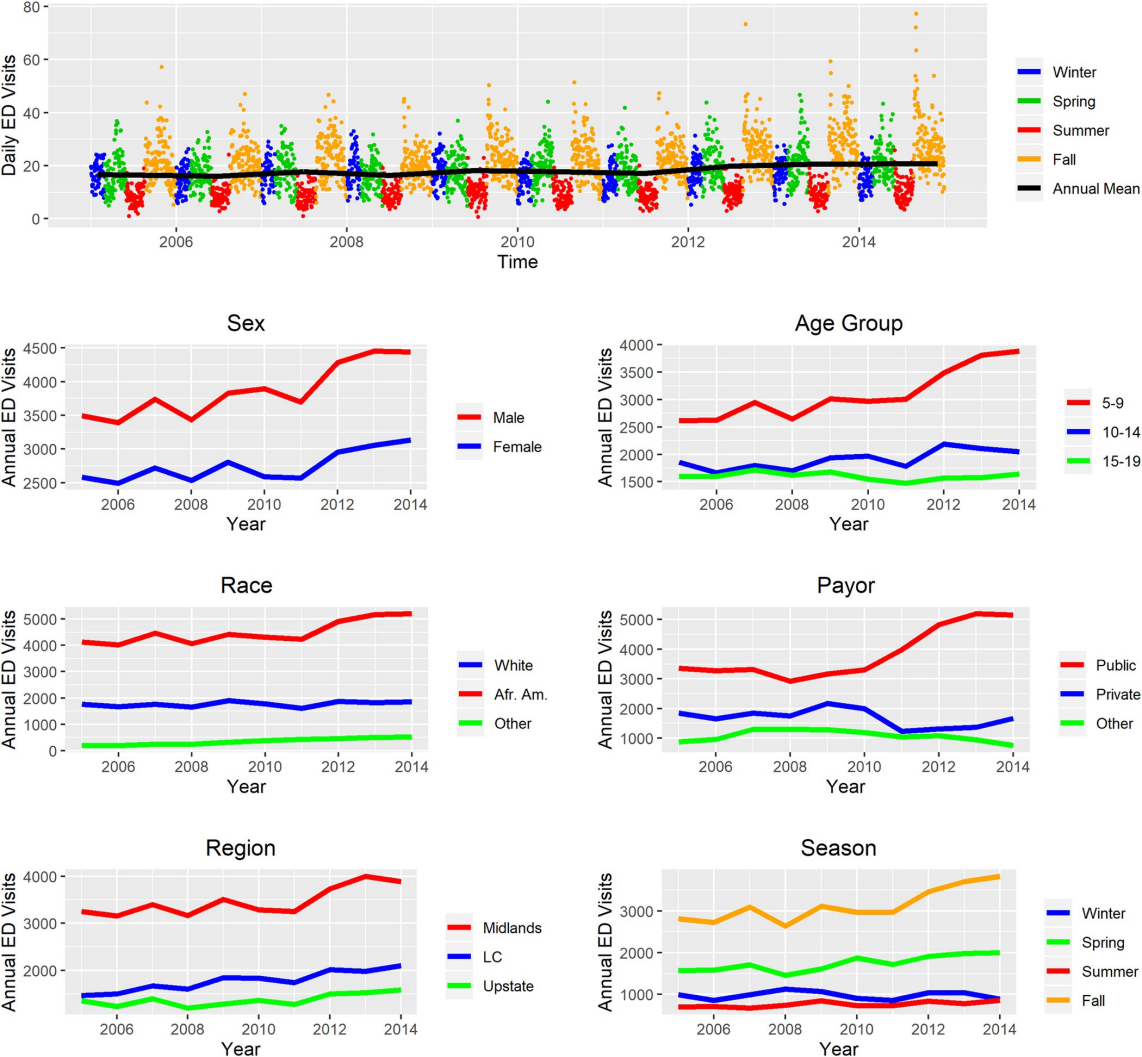
Asthma emergency department (ED) visits for ages 5–19 years from 2005 to 2014 in South Carolina by asthma season (winter: January 1 –February 28; spring: March 1 –May 31; summer: June 1 –August 19; fall: August 20 –December 31), sex, age group, race, payor, and geographic region.

The top center panel of Fig 3 shows the repeating seasonal pattern and a slowly increasing annual average of daily ED visits for asthma. In the remaining panels, large disparities in ED visits were observed for males, the youngest children (ages 5–9 years), African Americans, children on public insurance, children living in the Midlands, and seasonally in the fall asthma season. The fall asthma season had the greatest comparative burden, including a spike effect during the “back-to-school” period at the end of August and beginning of September. For most groups, disparities appeared to be increasing over the study period.

**Fig 4:**
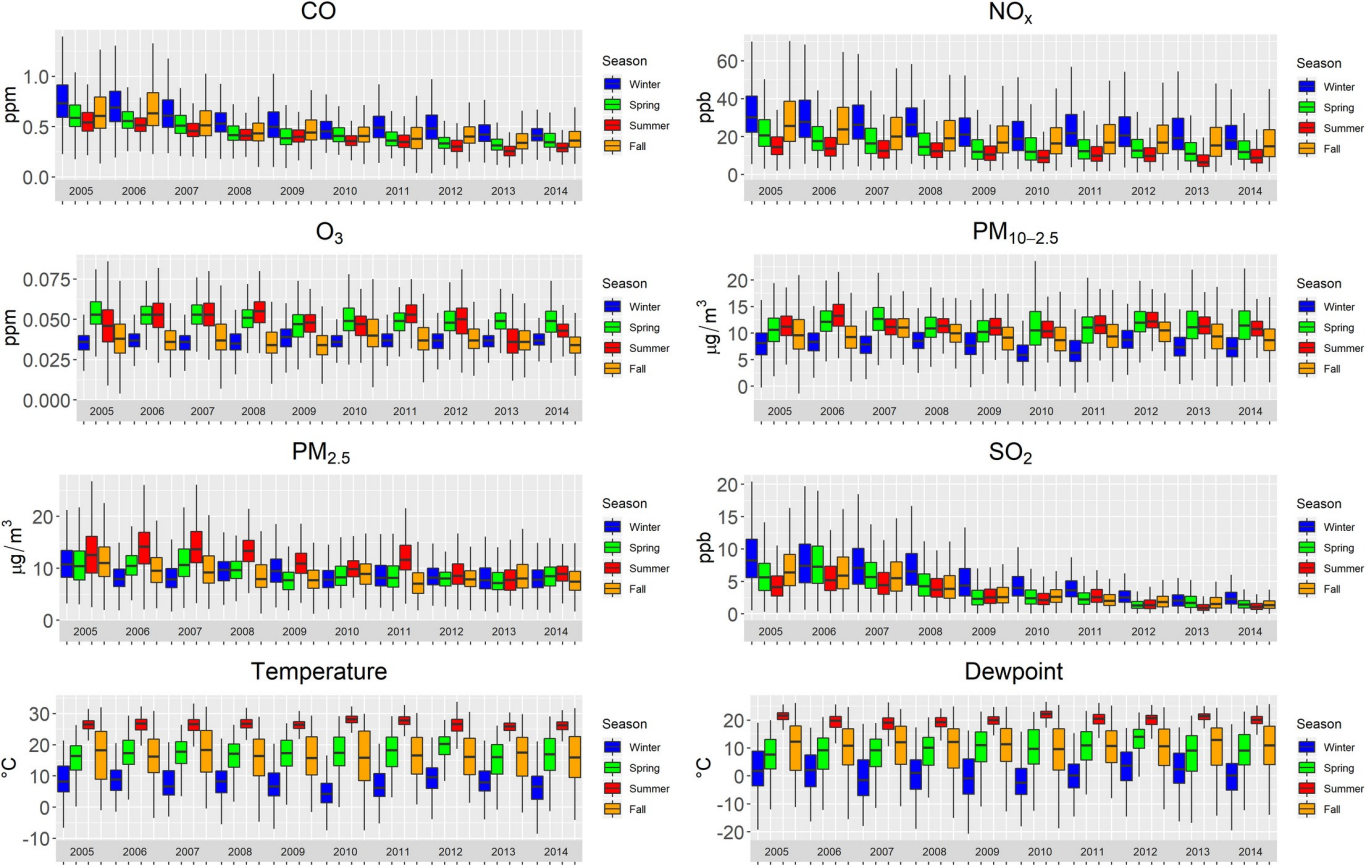
Estimated air pollution levels over time by pollutant and asthma season (winter: January 1 –February 28; spring: March 1 –May 31; summer: June 1 –August 19; fall: August 20 –December 31) in South Carolina from 2005 to 2014.

**Fig 5:**
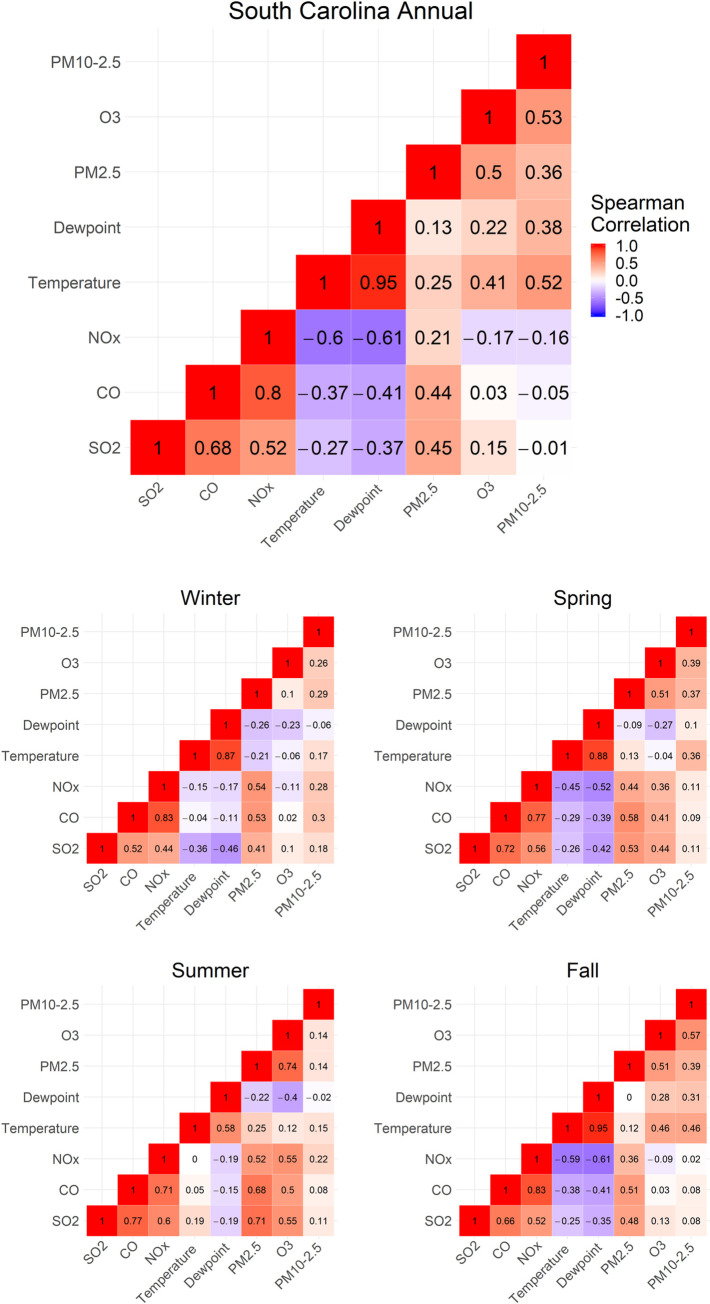
Heatmaps showing daily correlations between estimated pollutant levels in South Carolina for 2005–2014 by asthma season (winter: January 1 –February 28; spring: March 1 –May 31; summer: June 1 –August 19; fall: August 20 –December 31).

Except for O_3_ and potentially PM_10-2.5_, air pollutant levels seemed to decrease over the study period as shown in Fig 4. Each pollutant exhibited variation across seasons, with NO_x_, O_3_ and, to a lesser extent the PM measures, having the greatest between asthma season variation. Many correlations between the pollutants differed by asthma season for all years aggregated (Fig 5). For example, O_3_ was positively correlated with the NO_x_ in the spring and summer asthma seasons, but they were negatively correlated in fall and winter asthma seasons.

### 3.2 Statistical results

Results from the overall model of all SC ED visits for asthma among children showed increased odds of an ED visit from elevated levels of NO_x_ (OR_IQR_: 1.018, 95% CI: 1.002, 1.032) and PM_10-2.5_ (OR_IQR_: 1.054, 95% CI: 1.026, 1.063), controlling for other time-varying and invariant factors (Table 2). Results changed by asthma season, as air pollutants were not significantly associated with increased ED visit odds within the medium-burden winter and medium-high-burden spring asthma seasons. However, recognizing a small magnitude (often an order of magnitude lower than either’s independent association, respectively; results not shown) but statistically significant interaction between temperature and dewpoint temperature in each model, increased temperature was associated with asthma ED visits overall and in the spring and fall asthma seasons, respectively. Lower temperatures were significantly associated with increased ED visits in the low-burden summer asthma season. In the summer asthma season, there were statistically significant associations with two air pollutants: a negative association for NO_x_ (OR_IQR_: 0.954, 95% CI: 0.915, 0.991) and a positive association for PM_2.5_ (OR_IQR_: 1.162, 95% CI: 1.105, 1.222). In the high-burden fall, we found statistically significant associations with asthma ED visits that were positive for NO_x_ (OR_IQR_: 1.034, 95% CI: 1.009, 1.060), negative for PM_2.5_ (OR_IQR_: 0.970, 95% CI: 0.942, 0.998), and positive for PM_10-2.5_ (OR_IQR_: 1.144, 95% CI: 1.114, 1.177). Thus, the magnitude of the association between asthma ED visits and PM_10-2.5_ was thus nearly three times greater in fall relative to an entire year.

**Table 2:** Fully adjusted estimated odds ratios for interquartile range (IQR) increases in 3-day moving averages (3DMAs) of air pollutants (OR_IQR_), 95% credible intervals, and IQRs (overall and season-specific) for asthma emergency department (ED) visits among children ages 5–19 years in South Carolina (SC) from 2005 to 2014 by asthma season (winter: January 1 –February 28; spring: March 1 –May 31; summer: June 1 –August 19; fall: August 20 –December 31).

	Model				
Variable	Overall	Winter	Spring	Summer	Fall
	n = 66,092	n = 9,763	n = 17,414	n = 7,616	n = 31,299
**Pollutants**					
**NO**_**x**_	**1.018**	0.997	1.002	**0.954**	**1.034**
	(1.002,1.032)	(0.958,1.039)	(0.975,1.032)	(0.914,0.991)	(1.009,1.060)
	14.641 ppb	17.482 ppb	12.006 ppb	8.327 ppb	16.639 ppb
**O**_**3**_	1.001	1.000	0.997	1.010	0.999
	(0.987,1.018)	(0.988,1.012)	(0.976,1.012)	(0.988,1.043)	(0.983,1.016)
	0.017 ppm	0.008 ppm	0.012 ppm	0.015 ppm	0.014 ppm
**PM**_**10-2.5**_	**1.054**	1.015	0.983	1.012	**1.144**
	(1.026,1.063)	(0.975,1.057)	(0.951,1.015)	(0.975,1.052)	(1.114,1.177)
	4.479 μg/m^3^	3.813 μg/m^3^	4.617 μg/m^3^	3.343 μg/m^3^	4.329 μg/m^3^
**PM**_**2.5**_	1.014	1.007	0.999	**1.162**	**0.970**
	(0.997,1.031)	(0.966,1.050)	(0.963,1.034)	(1.105,1.222)	(0.942,0.998)
	4.599 μg/m^3^	4.052 μg/m^3^	4.124 μg/m^3^	5.441 μg/m^3^	4.734 μg/m^3^
**SO**_**2**_	0.998	1.025	1.012	N/A	0.994
	(0.984,1.012)	(0.985,1.071)	(0.981,1.044)		(0.976,1.013)
	3.727 ppb	4.881 ppb	3.723 ppb		3.618 ppb
**Weather**					
**Temp.** *	**1.123**	1.074	**1.147**	**0.895**	**1.204**
	(1.047,1.204)	(0.976,1.177)	(1.054,1.258)	(0.817,0.989)	(1.075,1.332)
	14.26°C	7.30°C	8.32°C	2.88°C	12.77°C
**Dewp.** *	N/A	1.092	1.091	N/A	N/A
		(0.964,1.227)	(0.975,1.228)		
		11.15°C	10.60°C		

Note: **Bold** font indicates statistical significance at α level 0.05. In each cell, the first line is the OR_IQR_ estimate, the second line is the 95% credible interval of the OR_IQR_, and the third line is the overall or season-specific IQR (depending on either overall or seasonal models).

Note: Time-invariant factors, such as sociodemographic characteristics, are controlled by the case-crossover design that contrasts environmental measures for ED visit case windows with those from referent windows, within individuals.

*Temperature and dewpoint temperature were included in a statistically significant interaction across all five models. The magnitude of effect was small, usually an order of magnitude smaller relative to the independent associations with either temperature or dewpoint temperature and asthma ED visits.

## 4.0 Discussion

Our first objective was to detail trends in asthma ED visits in SC. The trends we found are indicative of disparities over time and for specific subpopulations. Disparities generally increased for particular groups, including males, young children, African Americans, children on public insurance, and children living in the Midlands in SC from 2005 to 2014 (Fig 3). These results are partially consistent with national trends, as there are large disparities in asthma rates and outcomes across numerous factors including race, urban-rural status, socioeconomic status (SES), and others [59–61]. But the increasing disparities in SC over time are inconsistent with recent evidence that disparities may be plateauing in the US [62]. Furthermore, asthma ED visit disparities existed for males in the youngest age group (5–9 years) and among females in the oldest age group (15–19 years), which reinforces evidence that puberty and sex hormones likely play a role in asthma differences as children age [63, 64]. In addition, the specific subpopulation that visited the ED for asthma was notably different than the SC population of the same age 5-19-year group (Table 1). Sociodemographic and geographic disparities in asthma ED visits mirror broader health disparities in SC [65–69]. Further attention to the drivers of health disparities, including for asthma, are needed in places such as SC that may not follow national patterns.

To address our second objective of detailing the seasonal ambient environmental drivers of asthma ED visits, we introduced the concept of asthma seasons, defined by intra-annual periods of asthma ED visit burden. Our study location spanned both rural and urban areas, and we sought to avoid assuming that ambient air pollutants and weather patterns were necessarily the key seasonal influences. Overall, ED visits seemed to be increasing over time (Fig 3). ED visit patterns in SC seem to have four discernable asthma seasons that are similar to but still distinct from astronomical seasons, with particularly high burdens in the fall and spring asthma seasons (Figs 2 and 3). We were especially interested in the environmental drivers of fall and spring ED visits, given the relative disparities.

Asthma seasons should hypothetically differ by location based on geography, atmospheric chemistry, weather, land use, ecology, viral oscillation, and other factors, but not all acute health effects studies of air pollution adequately detail intra-annual temporal patterns of asthma outcomes locally. Those studies that do, for example in Shanghai, found that the intra-annual asthma burden similarly peaks over October, November, and December [9], but asthma ED visits there exhibit differing patterns in the remaining months of the year when contrasted with SC. Though not a study of environmental drivers, researchers identified a back-to-school peak when detailing asthma-related primary care provider visits by week and by subpopulation groups in Israel [70]. Back-to-school and other intra-annual patterns in asthma hospitalizations, in addition to exacerbation triggers, were detailed in a study in Texas [71]. Furthermore, when using SC’s asthma seasons, pollutants similarly varied by season (Figs 4 and 5).

We observed statistically significant, positive overall associations between both NO_x_ and PM_10_ and asthma ED visits in our overall model (Table 2), even though SC is generally considered a low air pollution state [72] and area, globally [73]. Previous studies have linked NO_x_ with overall asthma incidence [74], ED visits in cool seasons [3], and repeated visits [42]. Though we removed CO from statistical models because of its high collinearity with NO_x_, the main sources of both NO_x_ and CO are assumed to be emissions from fuel combustion [55]. As such, results suggest overall, season-invariant risks from fuel combustion in SC, even at relatively low overall pollutant levels.

That we found an overall, season-invariant association with PM_10-2.5_ and not PM_2.5_ in SC (Table 2) is somewhat surprising given the wide literature linking PM_2.5_ with asthma [4]. Neither daily nor long-term exposure to coarse PM were statistically significantly associated with respiratory outcomes among elderly Medicare patients [75, 76]. However, long-term exposure to coarse PM was linked to asthma in a nationwide pediatric Medicaid cohort [77]. Research has found that the commonly used CMAQ model has limitations in predicting ground-level PM_10_ from biogenic sources [78, 79]; consequently, studies that used CMAQ-only estimates for PM_10_ may have mischaracterized relationships with health outcomes. This study addresses the limitations by using estimates from the fusion model that provide more accurate estimates of daily PM_10_ [41], which may explain the improved ability for this analysis to detect health effects for this pollutant, particularly at lower levels.

We hypothesized that associations between asthma ED visits and air pollution varied by asthma season, and we generally found supporting evidence to corroborate the hypothesis. Relative to the overall model, the relationship between PM_10-2.5_ and asthma ED visits was stronger in the fall. While SC’s fall asthma season is, on average, neither consistently cool nor warm (Fig 4), others have linked increased PM_10_ to asthma ED visits in the cool season in nearby urban Atlanta, but not the warm season [8]. In urban Shanghai, associations between PM_10_ and asthma ED visits were null overall and within both warm and cool seasons, respectively. Given the results from this study, future research identifying the seasonally varying sources and PM_10_ components may help elucidate those that are key drivers of seasonal differences in asthma ED visits across urban-rural areas.

A potential seasonal allergenic component of PM_10_ could be respirable antigenic particles smaller than 10μm in diameter from larger pollen grains [80, 81]. Allergenic plants, such as ragweed, release seasonal pollen during the fall season in SC and other eastern and midwestern states, particularly in more rural and agricultural regions like the SC midlands. Allergenic particle levels tend to increase after rain events [21]. Agriculture and biomass burning, also common in the Midlands, should also be further studied as potentially important seasonally varying sources of PM_10_, as seasonal variation in PM_10_ fractions of elemental and organic carbon have been attributed to seasonal agricultural activity in other contexts [82]. However, allergens and other airborne irritants differ in mechanistic triggering of asthma exacerbations and may interact in complex ways to affect asthma pathogenesis across individuals [2], warranting additional research.

Summer had the lowest asthma burden in all of SC’s asthma seasons, yet two statistically significant air pollutant associations were found: a negative association with NO_x_ and a positive association with PM_2.5_. The estimated positive association with PM_2.5_, is a relationship that others have found in warm seasons in different locations [3, 83]. NO_x_, reaches its lowest and least variable levels in SC’s summer asthma season, and it may further be involved in complex interactions beyond the scope of this study. The primary source of NO_x_ is fuel combustion emissions [55] and NO_x_ is subsequently monitored mainly in SC’s urban areas characterized by high vehicle traffic density [56]. As such, there is little non-urban monitored data for calibrating modeled NO_x_ estimates in SC’s suburban and rural areas, which may be reflected by the fusion model’s somewhat mediocre NO_x_ performance relative to other pollutants [41]. Furthermore, air temperature had a relatively strong negative association, which is highly correlated with dewpoint temperature in summer in SC. The association with temperature, and by extension dewpoint temperature, indicates increased ED visit odds from cooler and drier conditions that are common in SC after weather fronts, as is similarly seen following thunderstorms [20].

By defining seasons relative to the outcome, we found that neither criteria pollutants nor weather were associated with asthma ED visits in a large portion of the year aligned with the winter and spring asthma seasons (January 1^st^–May 31^st^). Additional research is needed to tease apart drivers of ED visits in the medium-burden winter and medium-high-burden spring asthma seasons, respectively. However, it is important to contextualize the null associations with pollutants and weather covariates: outdoor ambient factors may simply play less of a role during these asthma seasons.

Common viruses, including influenza, usually peak in the coldest months, usually when people spend more time indoors [18]. Periods of high viral transmission encompass the winter and well into parts of the fall and spring in many places in the US [18]. In SC, temperatures are usually mild through fall, with appreciable cold temperatures often beginning only around early January each year, defined in this study as the winter asthma season. Others have found that daily viral transmission, annually peaking during the back-to-school portion of the fall and later in the winter, was the key predictor of asthma hospitalizations among children, and influenza prevalence was the key predictor of the annual winter surge in adult asthma hospitalizations [71]. We could not formally test the hypothesis that viruses are the main driver of the back-to-school surge in SC because we lacked viral transmission data. However, we saw a large spike each year around the time children went back to school in SC (Figs 2 and 3) that is suggestive of viral transmission as a potential contributor. Of additional interest, recent studies of the COVID-19 pandemic have found that asthma ED visit rates during this event were significantly reduced [84]. Researchers hypothesize that adoption of behaviors to reduce spread of the COVID-19 virus, such as wearing masks, are mechanisms that reduced transmission of many other types of viruses shown to exacerbate asthma [84, 85]. Given the mounting evidence of seasonal viral influences on asthma exacerbations, particularly during cold weather periods, future studies of the environmental drivers of seasonal differences in asthma should prioritize inclusion of such data.

Neither PM_2.5_ nor PM_10_ were significantly associated with ED visits in the spring asthma season. That no associations were found suggests that antigenic pollen particles from common spring-blooming plants such as grass and trees may have a minor or negligible role in asthma ED visits in a state like SC during its spring asthma season. This result contrasts with findings from many studies that have identified positive associations between asthma exacerbation events, such as ED visits, and both grass and tree pollen counts [12, 14, 16, 17, 19]. To more robustly assess hypotheses related to pollen in SC, inclusion of spatio-temporally varying pollen counts, species types, and particle size distributions need to be included.

### 4.1 Limitations

This research did not measure personal exposures, as it relied on interpolated census tract estimates of daily air pollutants and weather. Some air pollutants tend to vary greatly by location and across and within days (i.e., spatio-temporally). This research could not incorporate individual sensitivities to specific allergens, nor other individual asthma case-related information. We were unable to incorporate spatio-temporally resolved pollen counts or estimates, nor similar data for viruses. Time-varying indoor exposures such as environmental tobacco smoke (ETS) usage was not included. Similarly, children are not static in one location daily, but spend time indoors and outdoors at home, school, and many other locations. Consequently, there was potential for exposure misclassification. In addition, we found a few implausible protective associations that were potentially indicative of bias introduced by other complex environmental factors and synergisms, inaccurate estimates, uncontrolled confounders, or other factors. It indicated that even with relatively precise spatio-temporal resolution exposure estimates, teasing out independent effects of single environmental factors among many in a low-ambient air pollution using administrative health data setting remains highly challenging. It makes a case for incorporating environmental mixtures as opposed to single pollutant study designs. Finally, though we relied on 3-day moving averages guided by previous research findings, other lag structures may indicate significant associations between the same pollutants we identified by season, or different combinations.

### 4.2 Conclusion

We identified increasing asthma disparities across several socio-demographic factors in SC from 2005–2014, a departure from the plateauing national trend. We uniquely outlined the concept of asthma seasons that were defined by local intra-annual periods of relative burden. From such a perspective, the fall asthma season was the most burdensome for ED visits among children, followed by the spring and winter asthma seasons. The summer asthma season was the least burdensome. Results from Bayesian case-crossover analyses supported our hypothesis that associations between air pollution, weather, and asthma ED visits varied by asthma season. Across urban-rural areas characterized by generally low air pollution levels, there were acute health effects associated with NO_x_, particulate matter, and weather. But, associations differed by asthma season and PM size. With discretion, these results may be somewhat generalizable to geographically, demographically, and climatically-similar southern states. Our Bayesian methodology is reproducible for any location and can be tailored to any spatio-temporally-varying exposures for identifying and elucidating the acute health effects of local environmental exposures.
